# Physical Activity-Related Practices and Psychosocial Factors of Childcare Educators: A Latent Profile Analysis

**DOI:** 10.3390/children11040390

**Published:** 2024-03-25

**Authors:** Emma K. Adams, Andrea Nathan, Phoebe George, Stewart G. Trost, Jasper Schipperijn, Hayley Christian

**Affiliations:** 1 Telethon Kids Institute, University of Western Australia, Perth, WA 6009, Australia; emma.adams@telethonkids.org.au (E.K.A.);; 2 School of Population and Global Health, University of Western Australia, Perth, WA 6009, Australia; 3 School of Human Movement and Nutrition Sciences, University of Queensland, Brisbane, QLD 4072, Australia; s.trost@uq.edu.au; 4 Department of Sports Science Clinical Biomechanics, University of Southern Denmark, 5230 Odense, Denmark

**Keywords:** physical activity, childcare, latent profile analysis, educator

## Abstract

Limited research investigates early childhood education and care (ECEC) educators’ involvement in promoting physical activity. The aim was to identify distinct profiles based on physical activity-related practices and psychosocial factors in ECEC educators and examine how they relate to the amount of time allocated to children’s physical activity. A secondary analysis of educator-reported survey data from the Play Active study was undertaken. Educators (*n* = 532) reported on four practices and four psychosocial subscales adapted from the Environment and Policy Evaluation and Observation relating to the provision of physical activity in childcare. Latent profile analysis was used to identify distinct groups of educators based on their practices and psychosocial factors. Logistic regression analysed associations between latent profiles and educator-reported time provided for children’s physical activity. Five profiles of educators’ physical activity-related practices and psychosocial factors were identified. Profiles with higher practice scores also had higher psychosocial scores. Educators in profiles characterised by higher scores had greater odds of meeting the best practice guidelines for daily time allocated to children for total physical activity and energetic play. This study highlights interventions which address multiple educator behaviour change determinants to improve children’s physical activity in childcare.

## 1. Introduction

Being physically active is important for children’s health and development. It supports musculoskeletal strength, social and emotional wellbeing, cardiorespiratory fitness, and weight maintenance [1,2]. Early childhood is a critical period for establishing healthy movement behaviours, as childhood habits typically continue into later life and impact adult health outcomes [3]. However, a large proportion of young children do not meet daily physical activity recommendations [4,5,6].

Social-ecological theories of behaviour emphasise that health behaviours do not occur in a vacuum, but are influenced by multilevel factors and the physical and sociocultural environment [7,8]. The early childhood education and care (ECEC) setting is a key influence on young children’s physical activity [9]. Internationally, 90% of children aged 3 to 5 attend formal ECEC [10], and in Australia, 40% of children aged 5 and under attend ECEC [11]. In Australia, the National Quality Standards for ECEC sets a benchmark that “each child’s health and physical activity is supported and promoted” [12]. While these national standards exist, there is a lack of information, resources, and training to support educators to promote and provide physical activity to children in their care. Yet, research has identified that children spend up to 50% of their time in ECEC sitting [13] and are insufficiently active while in ECEC [14,15]. In recognition of the role of ECEC settings on young children’s physical activity [16,17], policies and practices focused on reducing children’s sedentary behaviours and increasing their physical activity levels are required [18,19,20]. However, implementation of such policies and practices is typically poor [19,21,22].

To improve the provision of physical activity for children in care, several key physical activity practices are necessary, including practices related to managers, supervisors, and educators; communicating with families; and the physical environment [23,24]. According to behaviour change theory [8], educators’ beliefs, self-efficacy, motivation, and support (referred to here as psychosocial factors) influence their physical activity-related practices, such as their role modelling, promoting, and planning for physical activity. Educator psychosocial factors and practices influence children’s physical activity as they are the gatekeepers to children’s activities and opportunities while in care [25,26,27,28].

There has been limited research to understand ECEC educator’s role in promoting children’s physical activity. A small body of qualitative research suggests that ECEC educators recognise their own practices, beliefs, and training can facilitate or impede their provision of physical activity for children in care [25,29,30,31]. Quantitative studies show that educators verbally promoting and joining in with children in physical activity is associated with higher levels of child physical activity [32,33,34,35,36,37]. Improving educators’ physical activity knowledge and self-efficacy can also increase children’s physical activity [38,39,40,41]. Yet, other research has found no associations between educator practices [42] or psychosocial factors [43] and children’s physical activity, and some ECEC educators believe their role is primarily care and supervision [31]. There is some evidence from school-based research that teachers’ promotion of physical activity is influenced by their self-efficacy, motivation, training, and support from colleagues [44,45,46]. In addition, teacher- and school-level socio-demographics (e.g., education, time worked in the sector, school socioeconomic status) have been associated with their physical activity practices [47,48,49]. Overall, findings from reviews suggest that the influence of ECEC educator practices on children’s physical activity is inconclusive [24,50,51]. Thus, further evidence is needed to confirm the relationship between ECEC educator physical activity-related practices and psychosocial factors and children’s physical activity levels [51], as well as whether there are certain educator physical activity-related practices and psychosocial factors that group together to affect children’s physical activity in ECEC.

In traditional variable-centred analyses (e.g., linear regression), there is an implicit assumption the individual factors have the same effect across the whole population. Person-centred approaches can overcome this limitation, as they identify subgroups of people who share attributes across a set of variables and therefore capture the unobserved heterogeneity of the sample [52]. Person-centred approaches better represent social-ecological theories because they specifically address the combination and accumulation of multiple factors [52]. Latent profile analysis (LPA) is one such person-centred approach. The profiles that emerge from the analysis represent a set of groupings of characteristics, and these profiles can then be used to investigate differences between profiles on other measures. LPA is thus a useful way to examine how the combination of ECEC educators’ physical activity-related practices and psychosocial factors support children’s physical activity provision in ECEC. Furthermore, identifying which variables cluster together can help inform more effective interventions, by developing strategies which simultaneously act on multiple factors.

To date, it appears person-centred analysis methods have not been used to examine combinations of ECEC educator physical activity-related characteristics, though there are some limited examples from studies conducted in the home and school settings. For instance, researchers have reported the clustering of physical and social factors related to home-based physical activity, with the more “healthful” clusters being positively associated with increased physical activity and sitting breaks in children [53]. Similarly, the profiles of school principals’ physical activity perceptions were associated with their involvement in the Comprehensive School Physical Activity Program (CSPAP) [54]. Specifically, principals characterised by above-average intrapersonal, interpersonal, and environmental perceptions of physical activity programs were more likely to have higher involvement in CSPAP, including promoting and organising physical activity opportunities.

Understanding how multiple ECEC educator factors group together can be used to identify patterns that are associated with increased opportunities for children to be active whilst attending care. Such findings can be used to inform ECEC physical activity interventions to assist with tailoring levels of support to meet the needs of different educators. Therefore, the aim of this study was to create educator profiles based on distinct physical activity practices and psychosocial factors and examine whether these relate to provision of time for physical activity to children in their care.

## 2. Materials and Methods

A secondary analysis of educator-reported survey data from the Play Active study was undertaken [55]. Play Active was a physical activity policy intervention with an overarching goal of increasing physical activity in young children attending ECEC. Play Active was implemented as a pragmatic cluster randomised trial in ECEC services in Perth, Western Australia. Full details of the Play Active protocol have been published elsewhere [55]. Ethics approval to conduct the study was obtained from The University of Western Australia Human Research Ethics Committee (RA/4/20/6120).

### 2.1. Participants

Play Active participant recruitment commenced following a launch event in December 2020. Interested ECEC services completed an online Expression of Interest form and were then contacted by the research team to screen for ineligibility and provide further study information. ECEC services were eligible if they were in the Perth and Peel metropolitan regions of Western Australia. Ineligibility criteria were a recent or anticipated change in management at the service (within three months), current service participation in another trial, services catering exclusively to children requiring specialist care, mobile preschools, family day care centres, and Department of Education and Communities preschools. Directors of eligible services provided consent to be involved in the study and gave contact details of their full- and part-time educators to be invited to participate in the evaluation. A total of 81 services and 573 educators participated in the baseline data collection.

### 2.2. Measures

#### 2.2.1. Practices and Psychosocial Factors

Educators completed surveys via the REDCap survey platform or via paper/PDF versions of the survey. The survey included 55 items across eight practice and psychosocial subscales relating to the provision of physical activity, sedentary behaviour, and screen time to children in their care [55]. Thirty-one items measured educator practices relating to planning and programming physical activity (e.g., I incorporate physical activity into room routines and transitions; *n* = 12), promoting physical activity (e.g., I talk with children about the importance of physical activity; *n* = 10), role modelling physical activity (e.g., I join children in physically active play; *n* = 6), and managing behaviour (e.g., I take away five or more minutes of active play time if children misbehave; *n* = 3). Additionally, 24 items measured educators’ psychosocial factors relating to physical activity beliefs (e.g., I enjoy being physically active with the children in my care; n = 6), self-efficacy (e.g., I feel able to provide children with opportunities for energetic play throughout the day; *n* = 7), motivation (e.g., I am motivated to provide children with opportunities for energetic play throughout the day; *n* = 7), and perceived social support from managers and other educators (e.g., I feel supported by management to promote young children’s physical activity). Survey items were obtained from the Nutrition and Physical Activity Self-Assessment for Child Care (NAP SACC) [56] and the Environment and Policy Evaluation and Observation as a Self-Report (EPAO-SR) [57], or were developed to specifically align with the Play Active physical activity policy recommendations and to measure key barriers and enablers identified in the policy development process [23,55,58]. The NAP SACC was developed to measure the best practices of ECEC educators relating to physical activity and sedentary behaviour [56]. The EPAO was developed based on evidence and expert review to measure the critical components of ECEC environments that support healthy weight, including those relating to educator practices regarding physical activity, sedentary time, and screen time [57,59]. The included measures have good reliability and comprehensively examine factors related to physical activity within the Australian ECEC context [58]. All practice items were measured on a six-point Likert scale from Never to Always, and all psychosocial items were measured on a seven-point Likert scale from Strongly Disagree to Strongly Agree. A summary of all items is available in Supplement File S1.

#### 2.2.2. Provision of Physical Activity

Educators reported the amount of daily time they allocated for indoor and outdoor physical activity (two items) on a seven-point ordinal scale (range ‘<30 min’–‘180+ min’). Responses were summed to provide an indicator of the total time allocated to physical activity. Educators also reported the amount of time allocated for energetic play (i.e., moderate-to-vigorous physical activity) on a five-point ordinal scale (range ‘<15 min’–‘60+ min’). Allocated time for total physical activity and energetic play were dichotomised into meeting or not meeting the best practice based on the Play Active physical activity policy recommendations of: (i) ≥180 min of total physical activity, (ii) ≥30 min of energetic play, and (iii) ≥180 min of total physical activity and ≥30 min of energetic play. Items were sourced from the EPAO and NAP SACC.

#### 2.2.3. Educator Characteristics

Educator characteristics, including year of birth, gender, highest level of education, length of time working in the sector, length of time working at their current service, usual hours of work, and the age/s of children they work with were collected in the survey. Educators also reported whether they had received professional development and training on children’s physical activity in the last two years.

### 2.3. Statistical Analyses

All items were coded so a higher response reflected a more positive outcome. The internal consistency of each subscale was confirmed using Cronbach’s alpha. Seven of eight subscales had acceptable alpha > 0.7, while the remaining subscale (managing behaviour) had alpha > 0.6 and was considered acceptable for these analyses (Supplementary Materials Tables S1 and S2).

Since the practice and psychosocial subscales had different response ranges, subscale scores were standardised to mean = 0.0 and SD = 1.0. The analysis used a bias-adjusted three-step approach [60]. To identify distinct groups of educator practices and psychosocial factors, the eight standardised subscale scores were entered into the LPA. Models with 1 through 10 classes were examined to determine the optimal number of profiles. Model selection was based on log-likelihood (LL), the Akaike Information Criterion (AIC), Consistent Akaike Information Criterion (CAIC), Bayesian Information Criterion (BIC), sample-size adjusted Bayesian Information Criterion (SABIC), entropy, interpretability, posterior probabilities, and profile size. A lower information criterion (LL, AIC, CAIC, BIC, SABIC) indicated a better fit, while higher entropy (>0.8) and higher average latent posterior probabilities (>0.9) indicated a better fit [52]. The characteristics of the emerging profiles were also considered to determine whether profiles were qualitatively and quantitatively distinct and made conceptual sense.

Second, educators were assigned to their most appropriate profile using proportional assignment adjusted for uncertainty when modelling profiles using the Bolck–Croon–Hagenaars (BCH) correction method [60]. Finally, the relationships between latent profiles and meeting best practice guidelines for the provision of physical activity to children in care were estimated with logistic regression models using the latent profile posterior probabilities and adjusted for educator characteristics. All data preparation and descriptive statistics were undertaken in SAS 9.4. The three-step LPA was undertaken in LatentGOLD 6.0 [61] using the cluster and step 3 procedures. All analyses adjusted for ECEC service-level clustering.

## 3. Results

Almost all respondents were female, and about half were certificate- or diploma-educated (training beyond high school but not at university level) (Table 1). Educators had worked within the ECEC sector for a median of 72 months and at their current service for a median of 19 months. Seventy percent of educators had previously received professional development on physical activity for children within the last two years.

### 3.1. Latent Profiles

**Figure 1:**
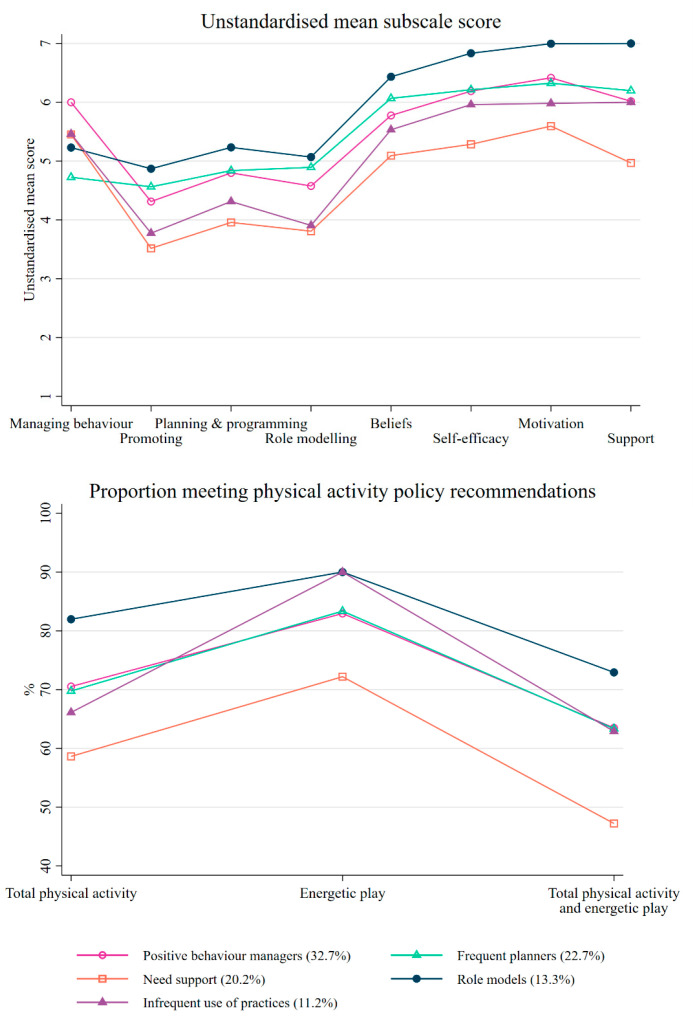
Unstandardised mean subscale scores and proportion of educators meeting phyiscal activity policy recommendations by latent profile.

The model with five profiles was selected as the best-fitting model (see Supplementary Materials File S2 for model fit statistics). For ease of interpretation, the unstandardised mean subscale scores were used to interpret the latent profiles (Figure 1). The standardised mean subscale scores are provided in Supplementary Materials Figure S1. The first profile, “Positive behaviour managers”, represented one-third of educators (32.7%) and was defined by the highest possible mean score for the managing behaviour subscale (mean = 6.0, Figure 1), meaning these educators ‘never’ used physical activity to manage children’s behaviour or removed physical activity as a response to misbehaviour. This profile had average scores across all other subscales. The second profile, “Frequent planners” (22.7% of educators), “very often” planned and role-modelled physical activity and “agreed” with the psychosocial factors, for example, that they were motivated to provide children with opportunities for energetic play. However, this profile also had the lowest observed score for the managing behaviour subscale, at 4.7. The third profile, “Need support” (20.2% of educators), had the lowest observed scores for almost all subscales, and notably, this profile only “somewhat agreed” that their management and colleagues supported young children’s physical activity. This profile also typically only “sometimes” to “often” implemented the physical activity-related practices and “somewhat agreed” with the other psychosocial factors. The fourth profile, “Role Models”, had the highest observed scores for almost all subscales and represented 13.3% of educators. These educators were “very often” using the practices and “agreed” or “strongly agreed” with all psychosocial factors. The final profile, “Infrequent use of practices” (11.2%), had lower scores for promoting, planning, role modelling, beliefs, and motivation, and average scores for managing behaviour, self-efficacy, and support subscales.

### 3.2. Association between Educator Profiles and Provision of Physical Activity

Latent profiles were significantly associated with meeting the best practice guideline for the provision of time for total physical activity (*p* = 0.050, Table 2). Specifically, the “Role models” profile had 3.2 times the odds (95% CI 1.5, 7.0) of meeting the policy recommended amount compared to the “Need support” profile. Overall, 82% of educators in the “Role models” profile met the best practice (Figure 1) compared to 59% of educators in the “Need support” profile.

The latent profiles were also associated with meeting the best practice guidelines for the combined provision of time for total physical activity and energetic play (*p* = 0.027). The “Role models” profile had 3.0 times the odds (95% CI 1.5, 6.1) of meeting the best practice, the “Positive behaviour managers” had 2.0 times the odds (95% CI 1.1, 3.4) of meeting the best practice, and the “Frequent planners” had 1.9 times the odds (95% CI 1.1, 3.6) of meeting the best practice compared to the “Need support” profile. Overall, 73% of educators in the “Role models” and 63% of educators in the “Positive behaviour managers” and “Frequent planners” profiles met the best practice compared to only 47% of educators in the “Need support” profile. Latent profiles were not significantly associated with meeting the best practice guideline for provision of time for energetic play (*p* = 0.070).

Logistic regression adjusted for educator age, educator education, age of children being worked with, length of time working in the sector, and length of time working at the current service.

## 4. Discussion

Person-centred approaches to factors related to the provision of physical activity for children in ECEC are scarce. This analysis identified that there were five distinct profiles of ECEC educators’ physical activity-related practices and psychosocial factors. Overall, profiles with higher practice scores also had higher psychosocial scores, and profiles with lower practice scores had lower psychosocial scores. Only 13% of educators were in the “Role models” subgroup, with high mean scores for practices that would provide children with many opportunities to be physically active. About half of educators (the “Frequent planners” and “Positive behaviour managers” subgroups) were characterised by scores on the subscales of practice and psychosocial factors within half a standard deviation above average, with only the managing behaviour subscale distinguishing the two most prevalent profiles. Thus, most educators had positive physical activity-related practices and beliefs, as well as high self-efficacy, motivation, and support relating to physical activity for children in ECEC. However, about one-third of educators were in one of the two profiles that had below-average practice and psychosocial scores (the “Need support” and “Infrequent use of practices” subgroups): one in five educators had scores one standard deviation below average for almost all subscales, and 11% of educators had promoting, planning, role modelling, and motivation subscales around 0.5 standard deviations below average. Educators in these profiles only sometimes to often used physical activity-related practices.

Previous ECEC research shows that the physical activity and sedentary opportunities educators provide may explain some of the variation in young children’s physical activity while attending childcare [32,62]. Specifically, positive associations have been reported between children’s physical activity and educators’ physical-activity-related practices, such as verbally encouraging and joining in with physical activity [26,32,33,34,35,36]. While these studies highlight that role modelling and physical activity promotion can have a positive impact on young children’s activity, overall, the evidence on the relationship between educator practices and children’s physical activity is limited and inconclusive [24,50], and there has been limited research which also examines the role of educators’ psychosocial factors in these relationships. This study found that educators with the highest practice and psychosocial scores (“Role models” profile) were more likely to provide the policy-recommended amounts of time for children to be physically active in ECEC compared to educators with low practice and psychosocial scores. Our findings strengthen previous research showing that educators’ physical activity practices are associated with the amount of time which they provide children to be active [25,29,30]. Our findings extend prior research highlighting that it is the combination of educator practices and psychosocial factors that can increase the amount of time educators provide for children to be active in ECEC. However, while socioecological models [8] and some prior research [27] indicate that the educator profiles identified in this study should correlate with children’s actual physical activity levels in ECEC, these relationships have not been explored here due the practical limitations of objectively measuring physical activity in all participating ECEC services. Further research is needed to understand the association between these educator profiles and children’s device-measured physical activity.

Around one in five educators were categorised into the “Need support” profile in terms of physical activity-related practices and psychosocial factors for supporting children’s physical activity in ECEC. This group was less likely to allocate the policy-recommended amounts of time for physical activity and energetic play. More research is needed in order to identify effective intervention strategies to improve educators’ practices and psychosocial factors to ensure children have sufficient opportunity for physical activity. For example, research has indicated that physical activity policy interventions to change educators’ practices may be better implemented when service managers view the policy as important, management and parents are supportive of the policy, and external resources (e.g., funding for professional development) to support policy implementation are accessible [21]. Positive social processes of encouragement and support can also improve beliefs regarding capabilities and motivation [63]. The “Need support” profile only somewhat agreed that they felt supported by managers and colleagues to promote physical activity to children in their care. Thus, strategies which also target broader social-ecological factors, including ECEC service directors, families, and government and non-government stakeholders [64], are needed to improve educators’ physical activity-related practices. The use of “champions” has also been identified as a strategy to increase support [65]. Identifying educators whose practices and attitudes are akin to the “Above average” group identified in this study could be a way to select champions of children’s physical activity in ECEC services to provide support to other educators.

While few educators had consistently high physical activity-related practices (the “Role models” profile), most profiles never or rarely used physical activity or screen time as a reward for good behaviour or removed physical activity if children misbehaved. This indicates that there are some widespread practices in ECEC settings that facilitate children having enjoyable and positive experiences with physical activity. Furthermore, this highlights that it is possible for there to be consistent practices within services and across the sector which all educators use to support children’s physical activity. Future research is needed in order to provide insight as to how other physical activity practices of educators could be more universally applied.

### Limitations

The educator physical activity promotion profiles identified in this study are specific to the sample, which was mostly female, post-secondary-school educated, and working in the ECEC sector for more than two years. Further research with larger samples is needed to identify whether similar profiles would be observed among other demographics, for example, among educators with only certificate-level education and who are new to the industry. Furthermore, ECEC services self-selected into the study and may have different physical activity-related profiles than the general ECEC sector. In addition, while educator subscales incorporated some items relating to ECEC manager and colleague support and engaging parents about their children’s physical activity at ECEC, social–ecological factors beyond ECEC were not included. Future research should consider the impact of the broader physical and economic environment on educator profiles and the allocation of time for children to be physically active while in care. Despite these limitations, the findings provide an innovative way to examine the combination of factors that can influence the amount of time educators provide for children to be physically active.

## 5. Conclusions

ECEC services are recognised as a key setting to target physical activity interventions to positively influence child health and development outcomes. Our findings highlight the importance of addressing educator behaviour change determinants, such as physical activity practices and psychosocial factors, together and not in isolation to improve the amount of time educators provide for children to be physically active. The results from this study can be used to inform intervention strategies, resulting in more effective interventions.

## Figures and Tables

**Table 1:** Educator characteristics.

	Median	IQR
Age in years (*n* = 568)	33.0	15.0
Length of time worked in childcare sector in months (*n* = 566)	72.0	97.0
Length of time worked in current service in months (*n* = 567)	19.0	42.0
Usual hours per week working in room in service (*n* = 559)	37.0	9.5
	*n*	%
Gender—Female (*n* = 572)	565	98.8
Highest schooling completed (*n* = 570)		
Year 12 or lower	123	21.6
Trade or diploma	291	51.1
University degree	156	27.4
Works with (*n* = 573)	390	68.1
Only toddlers (aged 1–2 years)	215	41.9
Only kindergarten children (aged 3–5 years)	148	28.8
Children aged 1–5	150	29.2
Received physical activity professional development in the last two years ^1^ (*n* = 518)	363	70.1
Meeting best practice guidelines for provision of physical activity		
Total physical activity (*n* = 532)	367	69.0
Energetic play (*n* = 525)	432	82.3
Total physical activity and energetic play (*n* = 525)	322	61.3

^1^ Received professional development on recommended amounts of daily physical activity and energetic play for young children or encouraging physical activity and energetic play in young children at least once in the last two years.

**Table 2:** Estimates (B (95% CI)) and odds (OR (95% CI)) of meeting policy recommendations for time allocated for physical activity.

	Meeting Total Physical Activity		Meeting Energetic Play		Meeting Both Guidelines	
	Β (95% CI)	OR (95% CI)	Β (95% CI)	OR (95% CI)	Β (95% CI)	OR (95% CI)
1. Positive behaviour managers	0.5 (−0.1, 1.1)	1.7 (0.9, 3.0)	0.6 (0.0, 1.3)	1.9 (1.0, 3.7)	0.7 (0.1, 1.2)	2.0 (1.1, 3.4)
2. Frequent planners	0.5 (−0.1, 1.1)	1.6 (0.9, 3.0)	0.7 (0.0, 1.4)	1.9 (1.0, 4.0)	0.7 (0.1, 1.3)	1.9 (1.1, 3.6)
3. Need support	Ref	Ref	Ref	Ref	Ref	Ref
4. Role models	1.2 (0.4, 1.9)	3.2 (1.5, 7.0)	0.9 (0.0, 1.7)	2.4 (1.0, 5.5)	1.1 (0.4, 1.8)	3.0 (1.5, 6.1)
5. Infrequent use of practices	0.3 (−0.4, 1.1)	1.4 (0.7, 2.9)	1.5 (0.3, 2.8)	4.6 (1.3, 16.2)	0.6 (−0.1, 1.4)	1.9 (0.9, 4.0)

## Data Availability

The data presented in this study are available on request from the corresponding author. The data are not publicly available due to specific ethical and privacy considerations.

## Supplementary Materials

The following supporting information can be downloaded at: https://www.mdpi.com/article/10.3390/children11040390/s1. Table S1. Description of physical activity practice survey items. Table S2. Description of attitude survey items. Table S3. Indicators of fit for LPA with one through ten profiles. Table S4. Posterior probabilities (mean (SD)) for each latent profile. Figure S1. Standardised mean subscale scores by latent profile.
