# Supplementary material for: Physical Activity-Related Practices and Psychosocial Factors of Childcare Educators: A Latent Profile Analysis
Source: Children (Basel). 2024 Mar 25;11(4):390. doi: 10.3390/children11040390 (PMC11049541; doi:10.3390/children11040390)
Supplement: Supplementary file 1 [file children-11-00390-s001.zip › children-2906191-supplementary.pdf]

children

MDPI

Supplementary Materials

Supplementary File S1 Description of survey items

Supplementary Table S1. Description of physical activity practice survey items.

Subscale	Item	Original Source	N	Mean	95% CI	Subscale alpha coefficient
Managing behaviour	I increase screen time as a reward for good behaviour or take it away for bad behaviour[1]	EPAO	536	1.2	1.1-1.3	0.611
	I take away 5 or more minutes of active play time if children misbehave[1]	EPAO	525	1.5	1.4-1.6	
	I increase outside time as a reward for good behaviour[1]	EPAO	508	2.0	1.9-2.2	
Promoting	I talk with children about the importance of physical activity	EPAO	524	4.2	4.1-4.3	0.827
	I praise children for being physically active	EPAO	520	4.5	4.4-4.6	
	I prompt children to increase their physical activity	EPAO	521	4.5	4.4-4.6	
	I prompt children to use portable play equipment during play time	EPAO	516	4.3	4.1-4.4	
	I prompt children to slow down their activity (e.g., running, jumping)[1]	EPAO	520	3.1	3.0-3.2	
	I encourage children to be active by talking about how fun it is to be physically active	EPAO	516	4.1	4.0-4.3	
	I talk with parents about their child's physical activity	EPAO	523	3.8	3.7-4.0	
	I encourage children to wear clothing and footwear that allows them to actively participate in physical activity	New	521	5.0	4.9-5.1	
	I encourage parents to create plenty of opportunities for their child to engage in physical activity and energetic play at home	New	515	3.7	3.5-3.8	
	I communicate regularly with families about physical activity experiences within our service	New	513	4.1	4.0-4.2	
Planning and programming	When screen time is offered, children are given the opportunity to do an alternative activity	EPAO	499	4.1	3.9-4.3	0.838

	I use cots, car seats and high chairs for their primary purpose only (cots for sleeping, car seats for vehicle travel, and high chairs for eating)	New	512	5.0	4.8-5.2	
	I incorporate physical activity into room routines and transitions	EPAO	522	4.2	4.1-4.3	
	I teach children about being physically active	EPAO	519	4.1	4.0-4.3	
	I make physical activity and energetic play part of children's everyday experiences		522	4.9	4.8-5.0	
	I program a range of learning experiences which encourage and use physical activity and energetic play	New	519	4.7	4.6-4.8	
	I provide opportunities for children to engage in discovery learning about the importance of physical activity and energetic play	New	516	4.3	4.2-4.4	
	I include physical activity as part of the assessment of children's physical and overall development	New	518	4.8	4.7-4.9	
	I provide children with adequate physical activity in poor weather	New	523	4.6	4.4-4.7	
	I provide age and developmentally appropriate structured physical activity for each child	New	523	4.7	4.6-4.8	
	I provide age and developmentally appropriate unstructured physical activity for each child	New	524	5.0	4.9-5.2	
	I provide opportunities for all children (including children with disabilities) to be physically active[2]	New	459	5.3	5.2-5.4	
Role modelling	I avoid sitting while supervising outside play	EPAO	534	4.0	3.9-4.2	0.767
	I join children in physically active play	EPAO	525	4.7	4.6-4.8	
	I join children in running and chasing games	EPAO	523	4.4	4.2-4.5	
	I show children that I enjoy being physically active at work	EPAO	522	4.5	4.4-4.6	
	I show children that I enjoy being physically active in my spare time	EPAO	517	3.7	3.6-3.8	
	I wear clothing and footwear that allows me to actively participate in physical activity	New	521	5.6	5.5-5.7	

Means and 95% confidence intervals (CI) are adjusted for clustering within ECEC services. All questions had response options 1=Never, 2=Rarely, 3=Sometimes, 4=Often, 5=Very often, 6=Always.

[1] A lower score is a better outcome. Item was reverse coded for the LPA.

[2] A not applicable response option was recoded to missing for these analyses.

Supplementary Table S2. Description of attitude survey items.

Sub-scale	Survey question	N	Mean	95% CI	Sub-scale alpha coefficient
Beliefs	I enjoy being physically active with the children in my care	509	6.3	6.2-6.4	0.735
	I find it easy to find creative ways for children to be physically active throughout the day	509	5.8	5.7-5.9	
	Weather extremes don't make it difficult for me to provide children with daily opportunities for physical activity	508	4.8	4.6-4.9	
	I believe parents are interested in their children's physical activity whilst in childcare	507	5.3	5.2-5.4	
	I believe physical activity is a learning experience for children	507	6.5	6.4-6.5	
	I believe educators should encourage children to use portable play equipment during play time	509	6.0	5.9-6.1	
Self-efficacy	I feel able to provide children with opportunities for indoor physical activity throughout the day	504	5.8	5.7-5.9	0.830
	I feel able to provide children with opportunities for outdoor physical activity throughout the day	505	6.2	6.1-6.3	
	I feel able to provide children with opportunities for energetic play throughout the day	504	6.0	5.9-6.1	
	I feel able to break up prolonged sitting and limit the total amount of time toddlers and kindergarten children spend sitting	504	6.0	5.9-6.1	
	I feel able to ensure children in my care don't have any sedentary screen time	505	6.2	6.1-6.4	
	I feel able to provide infants and babies with many opportunities for supervised interactive floor-based play throughout the day	491	6.1	6.0-6.2	
	I feel able to provide infants not yet mobile with many opportunities for tummy time throughout the day	490	6.1	6.0-6.2	
	I feel able to ensure cots, car seats, and high chairs are used for their primary purpose only (cots for sleeping, car seats for vehicle travel, and high chairs for eating)	494	6.4	6.3-6.5	

Motivation	I am motivated to provide children with opportunities for outdoor physical activity throughout the day	504	6.3	6.2-6.3	0.891
	I am motivated to provide children with opportunities for indoor physical activity throughout the day	503	6.1	6.0-6.2	
	I am motivated to provide children with opportunities for energetic play throughout the day	503	6.2	6.2-6.3	
	I am motivated to break up prolonged sitting and limit the total amount of time toddlers and kindergarten children spend sitting	499	6.3	6.2-6.3	
	I am motivated to ensure children in my care don't have any sedentary screen time	501	6.3	6.3-6.4	
	I am motivated to provide infants and babies with many opportunities for supervised interactive floor-based play throughout the day	491	6.3	6.2-6.3	
	I am motivated to provide infants not yet mobile with many opportunities for tummy time throughout the day	489	6.2	6.2-6.3	
	I am motivated to ensure cots, car seats, and high chairs are used for their primary purpose only (cots for sleeping, car seats for vehicle travel, and high chairs for eating)	493	6.4	6.3-6.5	
Support	I feel supported by management to promote young children's physical activity	502	6.0	5.9-6.1	0.898
	Other educators in this service support me in promoting young children's physical activity	504	5.9	5.8-6.0	
	I feel supported by management to break up prolonged sitting and limit the total amount of time young children spend sitting each day	500	6.1	5.9-6.2	
	Other educators in this service support me in breaking up prolonged sitting and limiting the total amount of time young children spend sitting each day	501	6.0	5.9-6.1	

Means and 95% confidence intervals (CI) are adjusted for clustering within ECEC services. All questions were developed specifically for this study.

All questions had response options 1=Strongly disagree, 2=Disagree, 3=Somewhat disagree, 4=Neither agree nor disagree, 5=Somewhat agree, 6=Agree, 7=Strongly agree.

Supplementary File S2 Latent profile analysis additional information

The BIC, AIC, CAIC, and SABIC all decreased with 1 through 6 latent profiles in the model. Entropy was high in all models, indicating high levels of classification precision. While fit indices did not clearly indicate the ideal number of profiles, there was limited improvement beyond six profiles. After further consideration of interpretability and cluster sizes, the model with five profiles was selected as the best model.

Supplementary Table S3. Indicators of fit for LPA with one through ten profiles.

N classes	LL	BIC	AIC	CAIC	SABIC	Entropy
1	-5898.780	11897.987	11829.561	11913.987	11847.198	1.000
2	-5254.187	10715.503	10574.374	10748.503	10610.751	0.860
3	-4829.968	9973.769	9759.936	10023.769	9815.054	0.893
4	-4528.172	9476.878	9190.343	9543.878	9264.200	0.900
**5**	**-4320.212**	**9167.662**	**8808.424**	**9251.662**	**8901.020**	**0.908**
6	-4175.965	8985.871	8553.930	9086.871	8665.266	0.904
7	-4133.558	9007.760	8503.116	9125.760	8633.192	0.893
8	-3972.131	8791.610	8214.263	8926.610	8363.079	0.911
9	-3975.910	8905.870	8255.820	9057.870	8423.376	0.910
10	-3877.347	8815.448	8092.695	8984.448	8278.991	0.912

Bold row indicates final model selection.

Supplementary Table S4. Posterior probabilities (mean (SD)) for each latent profile.

	Profile 1	Profile 2	Profile 3	Profile 4	Profile 5
1. Positive behaviour managers	**0.951 (0.096)**	0.011 (0.038)	0.026 (0.078)	0.004 (0.029)	0.008 (0.044)
2. Frequent planners	0.006 (0.050)	**0.943 (0.116)**	0.037 (0.093)	0.006 (0.045)	0.008 (0.043)
3. Need support	0.029 (0.093)	0.021 (0.075)	**0.937 (0.131)**	0.000 (0.000)	0.013 (0.062)
4. Role models	0.016 (0.066)	0.009 (0.034)	0.000 (0.000)	**0.975 (0.072)**	0.000 (0.000)
5. Infrequent use of practices	0.056 (0.102)	0.019 (0.058)	0.012 (0.031)	0.000 (0.000)	**0.912 (0.116)**

Bold cells are the average probability of a person being assigned to their profile.

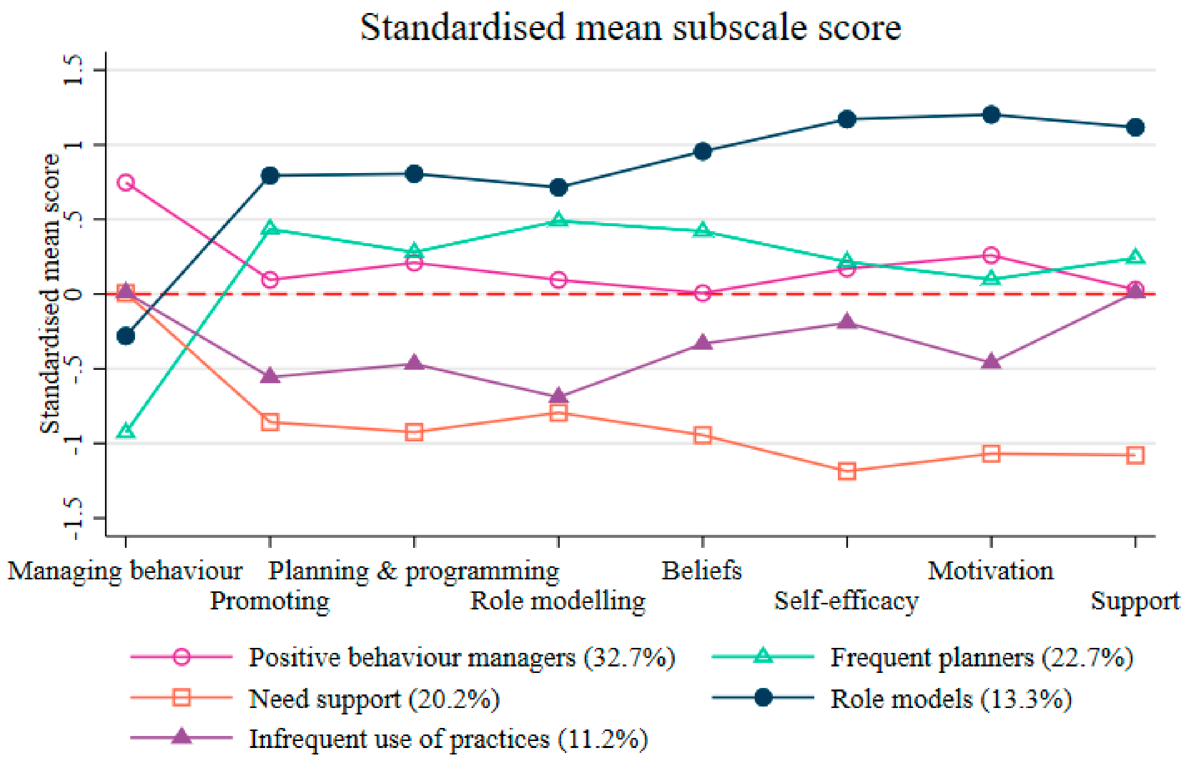

Supplementary Figure S1. Standardised mean subscale scores by latent profile.
